# Functional Glyconanomaterials

**DOI:** 10.3390/nano11102482

**Published:** 2021-09-24

**Authors:** Jose M. Palomo, M. Carmen Galan, Jose Manuel Garcia-Fernandez

**Affiliations:** 1 Department of Biocatalysis, Institute of Catalysis (CSIC), Marie Curie 2, 28049 Madrid, Spain; 2 School of Chemistry, University of Bristol, Cantock’s Close, Bristol BS8 1TS, UK; m.c.galan@bristol.ac.uk; 3 Instituto de Investigaciones Químicas (IIQ), CSIC, Universidad de Sevilla, 41092 Sevilla, Spain; jogarcia@iiq.csic.es

Nanotechnology provides a new array of techniques and platforms to study biological processes including glycosystems. Glyconanomaterials take advantage of the unique physical properties of the nanoscale, such as catalytic, photonic, electronic, or magnetic properties that are not seen in the bulk, as well as benefiting from the intrinsic properties of glycans, such as water solubility, biocompatibility, structural diversity, and targeting to specific receptors. Whilst metal, semiconductor, or carbon-based nanomaterials can confer their unique physical properties to glycans, glycans on the other hand provide these nanomaterials with an exceptional stability in water and biological buffers, with biocompatibility, and with both passive and active targeting properties. In addition, polysaccharides and glycodendrimers loaded onto these nanomaterials allow for fine-tuned control over the number and orientation of glycans on a nanosized architecture, permitting customized multivalent nanomaterials to be created.

Recent developments in the field have provided access to an advanced toolkit of synthetic nanomaterials and improved techniques to study such molecules at high resolution. Current advances in glycobiology research have demonstrated that glycosylated biomacromolecules play crucial roles in a wide range of important biological functions. Thus, the development of glycofunctionalized materials emulating or interfering in those processes, such as glycocarrier systems or glycoconjugate drugs, offer great potential for applications in a broad range of fields. The chemical diversity of carbohydrates can be further exploited for the development of new materials with controlled and precise chemical, physical, and biological properties.

In this framework, this Special Issue is devoted to compiling several papers on the design, fabrication, and utilization of glyconanomaterials for biomedical applications. Specifically, one research paper and four review papers are included in the Special Issue “Functional Glyconanomaterials”.

The paper of Rinaldi and coworkers [1] describes a simple methodology for synthesizing non-cytotoxic highly porous polysaccharide particles using a cross-linking strategy with different glycopolymers (dextran, hyaluronic acid). A nanopore glycomaterial was obtained which was successfully tested for efficient protein delivery under physiological conditions. After loading bovine serum albumin (BSA) and lysozyme onto the glyconanomaterial, the team demonstrated the sustained release of the two proteins and the dependence of the adsorption−release performance on pH, which have implications for medical applications and represent a possible way of tweaking the release profile.

Then, Galan and coworkers [2] in their review article describe the synthesis and potential application of carbon dots as photosensitiser nanomaterials for the specific detection and inactivation of different bacterial species. These carbon-based nanomaterials possess exceptional and tunable chemical and photoelectric properties as well as great chemical stability, high water solubility, low toxicity and excellent biocompatibility. In this review many examples of these nanomaterials, including glycan-coated carbon dots, were shown to be excellent candidates in antibacterial theranostic applications.

Another very interesting review by García Fernández and coworkers [3] is focused on the design and synthesis of multifunctionalized cyclodextrins (CDs) and the variety of functional glyconanomaterials empowered by the versatility of the CD component. These glycoconjugates have contributed to the understanding and exploitation of the interactions between multivalent glycodisplays and carbohydrate-binding proteins (lectins) and to improve the drug-loading and functional properties of nanomaterials through host–guest strategies.

Finally, two review articles have been focused mainly on the direct application of glyconanomaterials in biosensor applications [4,5].

Palomo and coworkers [4] described in their review article the advances on the development of glyconanomaterials focused on the detection of viruses, by using different approaches such as polysaccharide particles, oligosaccharide biosensors or protein biosensors. The design and application of glycomolecules conjugated with nanostructures, such as nanoparticles, have been emphasized as a novel antiviral therapy.

The paper of Tkac and coworkers describes the recent advances in the development of glycan nanobiosensors using diverse forms of nanomaterials (gold nanoparticles, quantum dots, magnetic nanoparticles, carbon nanoparticles, hybrid types of nanoparticles or proteins as nanoscaffolds). This review article is more focused on innovative immobilization strategies for the conjugation of a simple monosaccharide ligand with various types of nanomaterials for the subsequent detection of lectins, cells or viruses (e.g., influenza) to prove viability of the proof of concept.

We are confident that this Special Issue will provide the reader with an overall view of some of the latest prospects in this fast-evolving and cross-disciplinary field.
